# Methyl 3-*O*-α-l-fucopyranosyl β-d-glucopyran­oside tetra­hydrate

**DOI:** 10.1107/S1600536812041992

**Published:** 2012-10-20

**Authors:** Lars Eriksson, Göran Widmalm

**Affiliations:** a Department of Material and Environmental Chemistry, Arrhenius Laboratory, Stockholm University, SE-106 91 Stockholm, Sweden; b Department of Organic Chemistry, Arrhenius Laboratory, Stockholm University, SE-106 91 Stockholm, Sweden

## Abstract

The title compound, C_13_H_24_O_10_·4H_2_O, is the methyl glycoside of a disaccharide structural element present in the backbone of the capsular polysaccharide from *Klebsiella* K1, which contains only three sugars and a substituent in the polysaccharide repeating unit. The conformation of the title disaccharide is described by the glycosidic torsion angles ϕ_H_ = 51.1 (1)° and ψ_H_ = 25.8 (1)°. In the crystal, a number of O—H⋯O hydrogen bonds link the methyl glycoside and water mol­ecules, forming a three-dimensional network. One water mol­ecule is disordered over two positions with occupancies of 0.748 (4) and 0.252 (4).

## Related literature

For a background to capsular polysaccharides (CPS), see: Jansson *et al.* (1988 ▶); Erbing *et al.* (1976 ▶); Gloaguen *et al.* (1999 ▶); Cescutti *et al.* (2005 ▶). For details of the puckering analysis, see: Cremer & Pople (1975 ▶). For the synthesis, see: Baumann *et al.* (1988 ▶). For a related structure, see: Eriksson & Widmalm (2012 ▶).
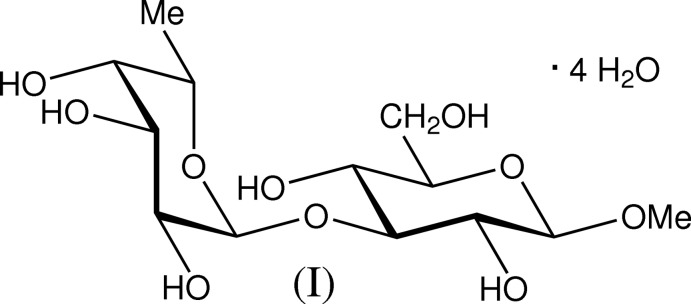


## Experimental

### 

#### Crystal data

C_13_H_24_O_10_·4H_2_O
*M* *_r_* = 412.39
Monoclinic, 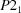

*a* = 9.6150 (2) Å
*b* = 7.1362 (1) Å
*c* = 13.9716 (2) Å
β = 100.1180 (18)°
*V* = 943.75 (3) Å^3^
*Z* = 2
Mo *K*α radiation
μ = 0.13 mm^−1^
*T* = 100 K
0.15 × 0.05 × 0.03 mm

#### Data collection

Oxford Xcalibur 3 diffractometer with Sapphire 3 CCD
Absorption correction: multi-scan (*CrysAlis RED*; Oxford Diffraction, 2008 ▶) *T*_min_ = 0.976, *T*_max_ = 0.996
37858 measured reflections
4836 independent reflections
4644 reflections with *I* > 2σ(*I*)
*R*_int_ = 0.030

#### Refinement

*R*[*F* ^2^ > 2σ(*F* ^2^)] = 0.028
*wR*(*F* ^2^) = 0.073
*S* = 1.08
4836 reflections
294 parameters
16 restraints
H atoms treated by a mixture of independent and constrained refinement
Δρ_max_ = 0.44 e Å^−3^
Δρ_min_ = −0.26 e Å^−3^

### 

Data collection: *CrysAlis CCD* (Oxford Diffraction, 2008 ▶); cell refinement: *CrysAlis CCD*; data reduction: *CrysAlis RED* (Oxford Diffraction, 2008); program(s) used to solve structure: *SHELXS97* (Sheldrick, 2008 ▶); program(s) used to refine structure: *SHELXL97* (Sheldrick, 2008 ▶); molecular graphics: *DIAMOND* (Brandenburg, 1999 ▶); software used to prepare material for publication: *PLATON* (Spek, 2009 ▶).

## Supplementary Material

Crystal structure: contains datablock(s) I, global. DOI: 10.1107/S1600536812041992/is5198sup1.cif

Structure factors: contains datablock(s) I. DOI: 10.1107/S1600536812041992/is5198Isup2.hkl

Additional supplementary materials:  crystallographic information; 3D view; checkCIF report

## Figures and Tables

**Table 1:** Hydrogen-bond geometry (Å, °)

*D*—H⋯*A*	*D*—H	H⋯*A*	*D*⋯*A*	*D*—H⋯*A*
O2*F*—H2*FA*⋯O*W*2	0.84	1.89	2.7326 (10)	176
O3*F*—H3*FA*⋯O2*F*	0.84	2.56	2.8696 (10)	103
O3*F*—H3*FA*⋯O*W*1^i^	0.84	1.92	2.7049 (10)	156
O4*F*—H4*FA*⋯O3*F* ^ii^	0.84	1.85	2.6836 (10)	172
O6*G*—H6*G*⋯O*W*3^iii^	0.84	1.86	2.6546 (11)	157
O2*G*—H2*G*1⋯O*W*1	0.84	1.87	2.6981 (10)	168
O4*G*—H4*G*1⋯O6*G* ^iv^	0.84	2.00	2.7884 (11)	155
O*W*1—H11⋯O2*F*	0.84 (2)	1.92 (2)	2.7522 (10)	172 (2)
O*W*1—H12⋯O4*F* ^i^	0.83 (2)	1.94 (2)	2.7646 (10)	178 (2)
O*W*2—H21⋯O6*G* ^iv^	0.81 (2)	2.09 (2)	2.8897 (10)	170 (2)
O*W*2—H22⋯O5*F* ^v^	0.86 (2)	1.91 (2)	2.7682 (11)	176 (2)
O*W*3—H31⋯O*W*4*A* ^i^	0.81 (2)	2.04 (2)	2.8424 (16)	176 (2)
O*W*3—H32⋯O7*M* ^vi^	0.87 (2)	2.00 (2)	2.8559 (13)	169 (2)
O*W*4*A*—H41*A*⋯O2*G*	0.83 (2)	2.10 (2)	2.8615 (16)	151 (3)
O*W*4*A*—H42*A*⋯O*W*2^vii^	0.83 (2)	2.11 (2)	2.9390 (15)	174 (4)

Symmetry codes: (i) 
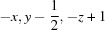
; (ii) 
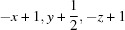
; (iii) 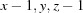; (iv) 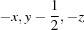; (v) 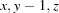; (vi) 
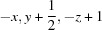
; (vii) 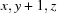.

**Fig. 1.**
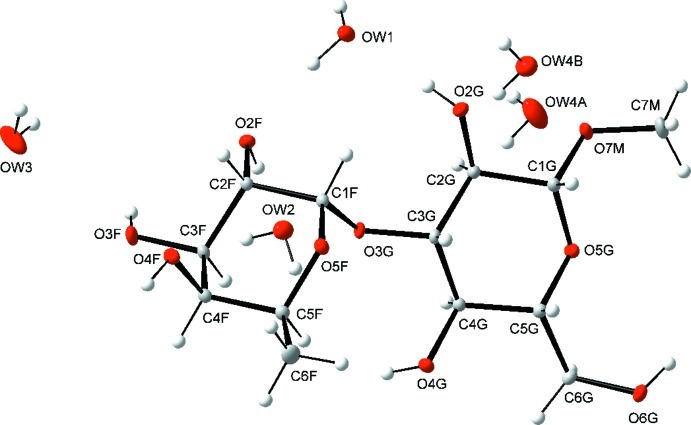
A view of the molecule with atom numbering scheme. The displacement ellipsoids are drawn at the 50% probability level for non-H atoms.

## supplementary crystallographic information

### Comment

Many polysaccharides are built of repeating units of oligosaccharides often having four or five sugar residues in their repeats. They may also carry substituents such as *O*-acetyl groups and/or pyruvic acids as for the *Klebsiella* capsular polysaccharides (CPS) termed S-53 and K1, respectively (Jansson *et al.*, 1988; Erbing *et al.*, 1976). The CPS of *Klebsiella* K1 contains a repeating unit consisting of → 4)-β-*D*-GlcpA-(2,3-Pyr)-(1→ 4)-α-*L*-Fucp-(1→ 3)-β-*D*-Glcp-(1→ (Erbing *et al.*, 1976). More recently the CPS S-53 from *Klebsiella* pneumoniae ATCC 31488 (Jansson *et al.*, 1988) was investigated, which, in addition, contained *O*-acetyl groups at positions 2 and 3 of the fucosyl residue. The disaccharide structural element is also present in the CPS produced by the thermophilic cynaobacterium *Mastigocladus laminosus* (Gloaguen *et al.*, 1999) and the exopolysaccharide from *Enterobacter amnigenus* (Cescutti *et al.*, 2005). The three-dimensional structure of the title compound, as a methyl glycoside model compound, represents an important part in these polysaccharides and may be used as a suitable starting point in modeling of the polymeric structures.

The major degrees of freedom in an oligosaccharide are the torsion angles φ_H_, ψ_H_, and ω. For the title compound (I) the two former are present at the glycosidic α-(1 → 3)-linkage. Furthermore, for the glucose residue the φ_H_ torsion angle is also important. The ω torsion angle describes the conformation of the hydroxymethyl group in the glucose residue. In the title compound both of the φ_H_ torsion angles in the structure are described by the *exo*-anomeric conformation with φ_H_ = 51.1 (1)° for the fucose residue and φ_H_ = 45.3 (1)° for the glucose residue (Fig. 1). The torsion angle conformation of ψ_H_ = 25.8 (1)°.

The conformation of the hydroxymethyl group is described by one of the three rotamers, *gauche-trans*, *gauche-gauche*, or *trans-gauche* with respect to the conformation of C6–O6 to C5–O5 and to C5–C4, respectively. In the present case the glucose residue has the *gt* conformation with ω = 77.02 (9)°, *i.e.*, shifted away somewhat from a canonical *gauche* conformation. Extensive water-water hydrogen bonding was observed (Table 1) for the four water molecules present in the crystal. Partial occupancy was observed for one of the water molecules, in a 3:1 relative ratio between OW4A and OW4B, with a distance of 0.90 Å in between the disordered water molecules.

The calculated Cremer & Pople (1975) parameters for the two different rings are: ring O5f → C5f [Q=0.5682 (9) Å, θ=172.92 (9) ° and φ=58.2 (7) °], ring O5g → C5g [Q=0.5822 (9) Å, θ=6.17 (9) ° and φ=279.5 (8) °]; consequently, the conformation of both rings can be described as chair-forms.

The crystal structure of methyl 3-*O*-α-*L*-fucopyranosyl α-*D*-galactopyranoside was recently determined (Eriksson & Widmalm, 2012), but in contrast to the title compound it did not crystallize as a hydrate. Two stereochemical differences are present between the compounds: firstly at the reducing end where the *O*-methyl group is located equatorially (β-configuration) in the title compound but axially (α-configuration) in methyl 3-*O*-α-*L*-fucopyranosyl α-*D*-galactopyranoside; secondly, and more interesting, the configuration at the C4 atom of the hexopyranose residue is different, equatorial in the title compound but axial in the previously investigated disaccharide, *i.e.*, from *gluco*- to *galacto*-configuration. For the φ_H_ dihedral angle the conformation at the glycosidic linkage is almost identical with φ_H_ = 51° herein and φ_H_ = 55° in methyl 3-*O*-α-*L*-fucopyranosyl α-*D*-galactopyranoside. However, the conformation at the ψ_H_ dihedral angle differs significantly with ψ_H_ = 26° herein, compared to ψ_H_ = -24° in the previously determined compound, *i.e.*, a difference of 50°. Whether the difference of *ca* 25° from an eclipsed conformation at the ψ_H_ dihedral angle represents an intrinsic difference at the glycosidic linkage between 3-*O*-substituted glucose and galactose residues, or is just due to packing/hydration effects, remains to be elucidated. We note that in water solution the ^13^C NMR glycosylation shifts (Δδ_C_), *i.e.*, differences in chemical shifts between the disaccharide and its constituent monosaccharides, for both the C1 and C3 atoms at the glycosidic linkage are lower by *ca* 1 p.p.m. in the title compound (Δδ_C_ ~7.2 p.p.m.) compared to those in methyl 3-*O*-α-*L*-fucopyranosyl α-*D*-galactopyranoside (Δδ_C_ ~8.3 p.p.m.) (Baumann *et al.*, 1988). These ^13^C NMR chemical shift differences may be related to different conformational preferences at the ψ_H_ dihedral angle.

### Experimental

The synthesis of the title compound was described by Baumann *et al.* (1988) in which the fucose and glucose residues have the *L* and *D* absolute configurations, respectively. The compound was crystallized by slow evaporation of a mixture of water and ethanol (1:1) at ambient temperature.

### Refinement

All hydrogen atoms, except those on the water molecules, were geometrically placed and constrained to ride on the parent atom. The C—H bond distances were set to 0.98 Å for CH_3_, 0.99 Å for CH_2_, 1.00 Å for CH. The O—H bond distance was set to 0.84 Å for OH groups. The *U*_iso_(H) = 1.5 *U*_eq_(C, O) for the CH_3_ and OH, while it was set to 1.2 *U*_eq_(C) for all other H atoms. One of the water positions, OW4 was disordered over two positions with the occupancy 0.748 (4) for OW4A and 0.252 (4) for OW4B. Using the non-merged dataset for refinement, the Flack parameter refined to *x* = 0.0 but the s.u. was estimated to 0.3. This low accuracy of *x* is a result of the absence of significant anomalous scattering effects, thus the value of the Flack parameter was not considered as meaningful, and the 2677 Friedel equivalents were included in the merging process (MERG 4 in *SHELXL*) for the final refinement. The absolute configuration of each sugar residue is known from the starting compounds used in the synthesis. The hydrogen atoms of the water molecule were located from difference density map, given *U*_iso_(H) = 1.5*U*_eq_(O) and in the refinement the d(O—H) and d(H..H) were restrained to retain the previously known geometry of the water molecule.

### Crystal data

**Table:**

C_13_H_24_O_10_·4H_2_O	*F*(000) = 444
*M**_r_* = 412.39	*D*_x_ = 1.451 Mg m^−^^3^
Monoclinic, *P*2_1_	Mo *K*α radiation, λ = 0.71073 Å
Hall symbol: P 2yb	Cell parameters from 26696 reflections
*a* = 9.6150 (2) Å	θ = 3.9–41.0°
*b* = 7.1362 (1) Å	µ = 0.13 mm^−^^1^
*c* = 13.9716 (2) Å	*T* = 100 K
β = 100.1180 (18)°	Prism, colourless
*V* = 943.75 (3) Å^3^	0.15 × 0.05 × 0.03 mm
*Z* = 2	

### Data collection

**Table:**

Oxford Xcalibur 3 diffractometer with Sapphire 3 CCD	4836 independent reflections
Radiation source: Enhance (Mo) X-ray Source	4644 reflections with *I* > 2σ(*I*)
Graphite monochromator	*R*_int_ = 0.030
Detector resolution: 16.5467 pixels mm^-1^	θ_max_ = 36.3°, θ_min_ = 4.0°
ω scans at different φ	*h* = −16→16
Absorption correction: multi-scan (*CrysAlis RED*; Oxford Diffraction, 2008)	*k* = −11→6
*T*_min_ = 0.976, *T*_max_ = 0.996	*l* = −23→23
37858 measured reflections	

### Refinement

**Table:**

Refinement on *F*^2^	Secondary atom site location: difference Fourier map
Least-squares matrix: full	Hydrogen site location: inferred from neighbouring sites
*R*[*F*^2^ > 2σ(*F*^2^)] = 0.028	H atoms treated by a mixture of independent and constrained refinement
*wR*(*F*^2^) = 0.073	*w* = 1/[σ^2^(*F*_o_^2^) + (0.0456*P*)^2^ + 0.0842*P*] where *P* = (*F*_o_^2^ + 2*F*_c_^2^)/3
*S* = 1.08	(Δ/σ)_max_ < 0.001
4836 reflections	Δρ_max_ = 0.44 e Å^−^^3^
294 parameters	Δρ_min_ = −0.26 e Å^−^^3^
16 restraints	Extinction correction: *SHELXL*, Fc^*^=kFc[1+0.001xFc^2^λ^3^/sin(2θ)]^-1/4^
Primary atom site location: structure-invariant direct methods	Extinction coefficient: 0.020 (4)

### Special details

**Table:**

Geometry. All e.s.d.'s (except the e.s.d. in the dihedral angle between two l.s. planes) are estimated using the full covariance matrix. The cell e.s.d.'s are taken into account individually in the estimation of e.s.d.'s in distances, angles and torsion angles; correlations between e.s.d.'s in cell parameters are only used when they are defined by crystal symmetry. An approximate (isotropic) treatment of cell e.s.d.'s is used for estimating e.s.d.'s involving l.s. planes.
Refinement. Refinement of *F*^2^ against ALL reflections. The weighted *R*-factor *wR* and goodness of fit *S* are based on *F*^2^, conventional *R*-factors *R* are based on *F*, with *F* set to zero for negative *F*^2^. The threshold expression of *F*^2^ > σ(*F*^2^) is used only for calculating *R*-factors(gt) *etc*. and is not relevant to the choice of reflections for refinement. *R*-factors based on *F*^2^ are statistically about twice as large as those based on *F*, and *R*- factors based on ALL data will be even larger.

### Fractional atomic coordinates and isotropic or equivalent isotropic displacement parameters (Å^2^)

**Table:**

	*x*	*y*	*z*	*U*_iso_*/*U*_eq_	Occ. (<1)
C1F	0.06137 (8)	0.74750 (12)	0.36039 (6)	0.00779 (13)	
H1F	−0.0206	0.8096	0.3826	0.009*	
C2F	0.12838 (8)	0.60944 (12)	0.43883 (6)	0.00787 (13)	
H2F	0.1488	0.6794	0.5017	0.009*	
C3F	0.26863 (8)	0.53512 (12)	0.41753 (6)	0.00798 (13)	
H3F	0.2504	0.4603	0.3561	0.010*	
C4F	0.36573 (8)	0.69748 (13)	0.40472 (6)	0.00811 (13)	
H4F	0.4554	0.6474	0.3877	0.010*	
C5F	0.29166 (9)	0.81952 (13)	0.32155 (6)	0.00935 (13)	
H5F	0.2719	0.7426	0.2608	0.011*	
O5F	0.15932 (7)	0.88703 (10)	0.34442 (5)	0.00936 (11)	
C6F	0.37691 (10)	0.98940 (15)	0.30347 (7)	0.01461 (16)	
H6FA	0.3243	1.0623	0.2495	0.022*	
H6FB	0.4672	0.9491	0.2869	0.022*	
H6FC	0.3945	1.0671	0.3622	0.022*	
O2F	0.03402 (7)	0.46140 (10)	0.45049 (5)	0.00979 (11)	
H2FA	0.0302	0.3855	0.4041	0.015*	
O3F	0.33526 (7)	0.41869 (11)	0.49451 (5)	0.01196 (12)	
H3FA	0.2829	0.3267	0.5004	0.018*	
O4F	0.39729 (7)	0.80024 (11)	0.49324 (5)	0.01022 (11)	
H4FA	0.4823	0.8340	0.5024	0.015*	
C1G	−0.32791 (9)	0.80214 (14)	0.11858 (6)	0.00982 (14)	
H1G	−0.3219	0.9420	0.1219	0.012*	
C2G	−0.23436 (8)	0.71631 (13)	0.20716 (6)	0.00919 (13)	
H2G	−0.2543	0.5790	0.2096	0.011*	
C3G	−0.07891 (8)	0.74567 (12)	0.20083 (6)	0.00806 (13)	
H3G	−0.0559	0.8822	0.2081	0.010*	
C4G	−0.04891 (8)	0.67751 (13)	0.10331 (6)	0.00803 (13)	
H4G	−0.0682	0.5399	0.0971	0.010*	
C5G	−0.14732 (9)	0.78118 (13)	0.02294 (6)	0.00859 (13)	
H5G	−0.1318	0.9192	0.0311	0.010*	
O5G	−0.28938 (6)	0.73757 (11)	0.03071 (4)	0.01060 (11)	
C6G	−0.12642 (9)	0.72355 (14)	−0.07751 (6)	0.01059 (14)	
H6G1	−0.0240	0.7129	−0.0782	0.013*	
H6G2	−0.1692	0.5985	−0.0927	0.013*	
O7M	−0.46601 (7)	0.74456 (12)	0.11844 (5)	0.01356 (13)	
C7M	−0.56733 (10)	0.8496 (2)	0.05233 (8)	0.0228 (2)	
H71	−0.5534	0.9837	0.0658	0.034*	
H72	−0.6629	0.8136	0.0605	0.034*	
H73	−0.5551	0.8234	−0.0145	0.034*	
O2G	−0.27031 (8)	0.80440 (12)	0.29016 (5)	0.01355 (13)	
H2G1	−0.2675	0.7254	0.3350	0.020*	
O3G	0.01081 (7)	0.64343 (10)	0.27542 (4)	0.00823 (11)	
O4G	0.09313 (7)	0.71213 (11)	0.09408 (5)	0.01101 (12)	
H4G1	0.1439	0.6219	0.1178	0.017*	
O6G	−0.18739 (7)	0.85281 (11)	−0.15098 (5)	0.01114 (12)	
H6G	−0.2758	0.8442	−0.1596	0.017*	
OW1	−0.23629 (7)	0.58785 (10)	0.45089 (5)	0.01226 (12)	
H11	−0.1538 (15)	0.546 (3)	0.4560 (12)	0.018*	
H12	−0.2840 (16)	0.500 (3)	0.4666 (12)	0.018*	
OW2	0.02672 (8)	0.22661 (11)	0.29518 (5)	0.01423 (13)	
H21	0.0697 (18)	0.276 (3)	0.2567 (12)	0.021*	
H22	0.0714 (19)	0.124 (2)	0.3125 (13)	0.021*	
OW3	0.53751 (9)	0.90725 (15)	0.79144 (8)	0.02575 (19)	
H31	0.4671 (19)	0.846 (3)	0.7728 (15)	0.039*	
H32	0.511 (2)	1.000 (3)	0.8244 (15)	0.039*	
OW4A	−0.28298 (13)	1.20268 (19)	0.26647 (12)	0.0277 (4)	0.748 (4)
H41A	−0.297 (3)	1.097 (3)	0.288 (2)	0.042*	0.748 (4)
H42A	−0.196 (2)	1.217 (5)	0.276 (2)	0.042*	0.748 (4)
OW4B	−0.3277 (4)	1.2108 (6)	0.3181 (2)	0.0192 (9)	0.252 (4)
H41B	−0.269 (7)	1.128 (10)	0.313 (6)	0.029*	0.252 (4)
H42B	−0.307 (7)	1.261 (10)	0.371 (3)	0.029*	0.252 (4)

### Atomic displacement parameters (Å^2^)

**Table:**

	*U*^11^	*U*^22^	*U*^33^	*U*^12^	*U*^13^	*U*^23^
C1F	0.0070 (3)	0.0093 (3)	0.0069 (3)	0.0005 (3)	0.0005 (2)	0.0001 (2)
C2F	0.0065 (3)	0.0099 (3)	0.0070 (3)	0.0003 (2)	0.0007 (2)	0.0009 (2)
C3F	0.0061 (3)	0.0088 (3)	0.0088 (3)	0.0007 (2)	0.0007 (2)	0.0010 (3)
C4F	0.0063 (3)	0.0097 (3)	0.0081 (3)	−0.0002 (2)	0.0009 (2)	0.0001 (2)
C5F	0.0080 (3)	0.0111 (3)	0.0090 (3)	0.0001 (3)	0.0015 (2)	0.0014 (3)
O5F	0.0076 (2)	0.0089 (3)	0.0114 (3)	0.0000 (2)	0.00116 (19)	0.0009 (2)
C6F	0.0130 (3)	0.0144 (4)	0.0165 (4)	−0.0030 (3)	0.0028 (3)	0.0058 (3)
O2F	0.0081 (2)	0.0116 (3)	0.0100 (2)	−0.0015 (2)	0.00258 (19)	0.0013 (2)
O3F	0.0076 (2)	0.0121 (3)	0.0152 (3)	0.0007 (2)	−0.0007 (2)	0.0063 (2)
O4F	0.0071 (2)	0.0134 (3)	0.0097 (2)	−0.0020 (2)	0.00025 (18)	−0.0024 (2)
C1G	0.0070 (3)	0.0138 (3)	0.0086 (3)	0.0012 (3)	0.0013 (2)	0.0021 (3)
C2G	0.0078 (3)	0.0125 (3)	0.0072 (3)	0.0013 (3)	0.0013 (2)	0.0014 (3)
C3G	0.0075 (3)	0.0095 (3)	0.0067 (3)	0.0011 (3)	0.0000 (2)	0.0014 (3)
C4G	0.0067 (3)	0.0101 (3)	0.0071 (3)	0.0001 (2)	0.0007 (2)	0.0007 (2)
C5G	0.0080 (3)	0.0105 (3)	0.0073 (3)	−0.0002 (2)	0.0012 (2)	0.0004 (3)
O5G	0.0069 (2)	0.0165 (3)	0.0081 (2)	−0.0010 (2)	0.00048 (18)	−0.0002 (2)
C6G	0.0121 (3)	0.0125 (3)	0.0070 (3)	0.0010 (3)	0.0012 (2)	0.0007 (3)
O7M	0.0058 (2)	0.0206 (3)	0.0140 (3)	0.0013 (2)	0.0009 (2)	0.0068 (3)
C7M	0.0092 (3)	0.0371 (6)	0.0211 (4)	0.0065 (4)	0.0002 (3)	0.0131 (4)
O2G	0.0137 (3)	0.0198 (3)	0.0079 (2)	0.0055 (3)	0.0039 (2)	0.0018 (2)
O3G	0.0086 (2)	0.0092 (3)	0.0059 (2)	0.0017 (2)	−0.00137 (18)	−0.0001 (2)
O4G	0.0066 (2)	0.0156 (3)	0.0111 (2)	0.0003 (2)	0.00206 (19)	0.0016 (2)
O6G	0.0108 (2)	0.0142 (3)	0.0076 (2)	−0.0013 (2)	−0.00058 (19)	0.0023 (2)
OW1	0.0111 (3)	0.0116 (3)	0.0148 (3)	0.0017 (2)	0.0043 (2)	0.0029 (2)
OW2	0.0165 (3)	0.0106 (3)	0.0163 (3)	0.0008 (2)	0.0047 (2)	0.0010 (2)
OW3	0.0134 (3)	0.0249 (4)	0.0370 (5)	0.0026 (3)	−0.0009 (3)	−0.0141 (4)
OW4A	0.0169 (5)	0.0176 (6)	0.0475 (9)	0.0016 (4)	0.0029 (5)	0.0056 (5)
OW4B	0.0190 (14)	0.0227 (17)	0.0162 (14)	0.0014 (12)	0.0039 (11)	−0.0007 (12)

### Geometric parameters (Å, º)

**Table:**

C1F—O3G	1.4122 (10)	C3G—H3G	1.0000
C1F—O5F	1.4149 (11)	C4G—O4G	1.4158 (10)
C1F—C2F	1.5292 (11)	C4G—C5G	1.5265 (11)
C1F—H1F	1.0000	C4G—H4G	1.0000
C2F—O2F	1.4205 (11)	C5G—O5G	1.4236 (10)
C2F—C3F	1.5261 (11)	C5G—C6G	1.5094 (12)
C2F—H2F	1.0000	C5G—H5G	1.0000
C3F—O3F	1.4197 (10)	C6G—O6G	1.4272 (11)
C3F—C4F	1.5182 (12)	C6G—H6G1	0.9900
C3F—H3F	1.0000	C6G—H6G2	0.9900
C4F—O4F	1.4240 (11)	O7M—C7M	1.4311 (12)
C4F—C5F	1.5248 (12)	C7M—H71	0.9800
C4F—H4F	1.0000	C7M—H72	0.9800
C5F—O5F	1.4479 (11)	C7M—H73	0.9800
C5F—C6F	1.5094 (13)	O2G—H2G1	0.8400
C5F—H5F	1.0000	O4G—H4G1	0.8400
C6F—H6FA	0.9800	O6G—H6G	0.8400
C6F—H6FB	0.9800	OW1—H11	0.839 (14)
C6F—H6FC	0.9800	OW1—H12	0.830 (15)
O2F—H2FA	0.8400	OW2—H21	0.814 (15)
O3F—H3FA	0.8400	OW2—H22	0.862 (15)
O4F—H4FA	0.8400	OW3—H31	0.810 (16)
C1G—O7M	1.3896 (11)	OW3—H32	0.868 (16)
C1G—O5G	1.4200 (11)	OW4A—OW4B	0.904 (4)
C1G—C2G	1.5247 (11)	OW4A—H41A	0.835 (19)
C1G—H1G	1.0000	OW4A—H42A	0.826 (18)
C2G—O2G	1.4143 (11)	OW4A—H41B	0.83 (9)
C2G—C3G	1.5271 (11)	OW4B—H41A	0.98 (3)
C2G—H2G	1.0000	OW4B—H41B	0.83 (2)
C3G—O3G	1.4306 (10)	OW4B—H42B	0.81 (2)
C3G—C4G	1.5213 (11)		
			
O3G—C1F—O5F	112.20 (6)	O2G—C2G—C1G	107.01 (7)
O3G—C1F—C2F	107.64 (7)	O2G—C2G—C3G	111.67 (7)
O5F—C1F—C2F	110.98 (6)	C1G—C2G—C3G	109.98 (7)
O3G—C1F—H1F	108.6	O2G—C2G—H2G	109.4
O5F—C1F—H1F	108.6	C1G—C2G—H2G	109.4
C2F—C1F—H1F	108.6	C3G—C2G—H2G	109.4
O2F—C2F—C3F	111.59 (7)	O3G—C3G—C4G	107.73 (7)
O2F—C2F—C1F	111.36 (6)	O3G—C3G—C2G	111.03 (7)
C3F—C2F—C1F	111.08 (7)	C4G—C3G—C2G	110.46 (7)
O2F—C2F—H2F	107.5	O3G—C3G—H3G	109.2
C3F—C2F—H2F	107.5	C4G—C3G—H3G	109.2
C1F—C2F—H2F	107.5	C2G—C3G—H3G	109.2
O3F—C3F—C4F	109.32 (6)	O4G—C4G—C3G	111.31 (7)
O3F—C3F—C2F	110.63 (7)	O4G—C4G—C5G	109.37 (7)
C4F—C3F—C2F	109.91 (7)	C3G—C4G—C5G	108.26 (7)
O3F—C3F—H3F	109.0	O4G—C4G—H4G	109.3
C4F—C3F—H3F	109.0	C3G—C4G—H4G	109.3
C2F—C3F—H3F	109.0	C5G—C4G—H4G	109.3
O4F—C4F—C3F	109.40 (7)	O5G—C5G—C6G	107.31 (7)
O4F—C4F—C5F	111.56 (7)	O5G—C5G—C4G	108.48 (7)
C3F—C4F—C5F	108.13 (6)	C6G—C5G—C4G	112.66 (7)
O4F—C4F—H4F	109.2	O5G—C5G—H5G	109.4
C3F—C4F—H4F	109.2	C6G—C5G—H5G	109.4
C5F—C4F—H4F	109.2	C4G—C5G—H5G	109.4
O5F—C5F—C6F	107.09 (7)	C1G—O5G—C5G	113.24 (6)
O5F—C5F—C4F	109.44 (7)	O6G—C6G—C5G	112.81 (8)
C6F—C5F—C4F	113.06 (7)	O6G—C6G—H6G1	109.0
O5F—C5F—H5F	109.1	C5G—C6G—H6G1	109.0
C6F—C5F—H5F	109.1	O6G—C6G—H6G2	109.0
C4F—C5F—H5F	109.1	C5G—C6G—H6G2	109.0
C1F—O5F—C5F	115.83 (7)	H6G1—C6G—H6G2	107.8
C5F—C6F—H6FA	109.5	C1G—O7M—C7M	112.83 (8)
C5F—C6F—H6FB	109.5	O7M—C7M—H71	109.5
H6FA—C6F—H6FB	109.5	O7M—C7M—H72	109.5
C5F—C6F—H6FC	109.5	H71—C7M—H72	109.5
H6FA—C6F—H6FC	109.5	O7M—C7M—H73	109.5
H6FB—C6F—H6FC	109.5	H71—C7M—H73	109.5
C2F—O2F—H2FA	109.5	H72—C7M—H73	109.5
C3F—O3F—H3FA	109.5	C2G—O2G—H2G1	109.5
C4F—O4F—H4FA	109.5	C1F—O3G—C3G	114.75 (7)
O7M—C1G—O5G	107.29 (7)	C4G—O4G—H4G1	109.5
O7M—C1G—C2G	108.04 (7)	C6G—O6G—H6G	109.5
O5G—C1G—C2G	111.41 (7)	H11—OW1—H12	105.3 (16)
O7M—C1G—H1G	110.0	H21—OW2—H22	105.5 (16)
O5G—C1G—H1G	110.0	H31—OW3—H32	106.1 (18)
C2G—C1G—H1G	110.0		
			
O3G—C1F—C2F—O2F	−52.73 (8)	O2G—C2G—C3G—O3G	69.76 (9)
O5F—C1F—C2F—O2F	−175.86 (7)	C1G—C2G—C3G—O3G	−171.59 (7)
O3G—C1F—C2F—C3F	72.31 (8)	O2G—C2G—C3G—C4G	−170.80 (7)
O5F—C1F—C2F—C3F	−50.82 (9)	C1G—C2G—C3G—C4G	−52.15 (10)
O2F—C2F—C3F—O3F	−59.67 (9)	O3G—C3G—C4G—O4G	−61.52 (9)
C1F—C2F—C3F—O3F	175.42 (7)	C2G—C3G—C4G—O4G	177.06 (7)
O2F—C2F—C3F—C4F	179.51 (6)	O3G—C3G—C4G—C5G	178.25 (7)
C1F—C2F—C3F—C4F	54.60 (9)	C2G—C3G—C4G—C5G	56.82 (9)
O3F—C3F—C4F—O4F	−58.47 (8)	O4G—C4G—C5G—O5G	177.31 (7)
C2F—C3F—C4F—O4F	63.14 (8)	C3G—C4G—C5G—O5G	−61.25 (9)
O3F—C3F—C4F—C5F	179.85 (7)	O4G—C4G—C5G—C6G	58.65 (10)
C2F—C3F—C4F—C5F	−58.54 (8)	C3G—C4G—C5G—C6G	−179.91 (7)
O4F—C4F—C5F—O5F	−61.09 (9)	O7M—C1G—O5G—C5G	−177.77 (7)
C3F—C4F—C5F—O5F	59.25 (9)	C2G—C1G—O5G—C5G	−59.71 (10)
O4F—C4F—C5F—C6F	58.17 (9)	C6G—C5G—O5G—C1G	−173.98 (7)
C3F—C4F—C5F—C6F	178.52 (7)	C4G—C5G—O5G—C1G	64.03 (9)
O3G—C1F—O5F—C5F	−65.99 (9)	O5G—C5G—C6G—O6G	77.02 (9)
C2F—C1F—O5F—C5F	54.48 (9)	C4G—C5G—C6G—O6G	−163.63 (7)
C6F—C5F—O5F—C1F	177.74 (7)	O5G—C1G—O7M—C7M	−73.36 (11)
C4F—C5F—O5F—C1F	−59.37 (9)	C2G—C1G—O7M—C7M	166.41 (9)
O7M—C1G—C2G—O2G	−68.77 (9)	O5F—C1F—O3G—C3G	−69.16 (8)
O5G—C1G—C2G—O2G	173.62 (7)	C2F—C1F—O3G—C3G	168.45 (6)
O7M—C1G—C2G—C3G	169.75 (8)	C4G—C3G—O3G—C1F	144.26 (7)
O5G—C1G—C2G—C3G	52.15 (10)	C2G—C3G—O3G—C1F	−94.67 (8)

### Hydrogen-bond geometry (Å, º)

**Table:**

*D*—H···*A*	*D*—H	H···*A*	*D*···*A*	*D*—H···*A*
O2*F*—H2*FA*···O*W*2	0.84	1.89	2.7326 (10)	176
O3*F*—H3*FA*···O2*F*	0.84	2.56	2.8696 (10)	103
O3*F*—H3*FA*···O*W*1^i^	0.84	1.92	2.7049 (10)	156
O4*F*—H4*FA*···O3*F*^ii^	0.84	1.85	2.6836 (10)	172
O6*G*—H6*G*···O*W*3^iii^	0.84	1.86	2.6546 (11)	157
O2*G*—H2*G*1···O*W*1	0.84	1.87	2.6981 (10)	168
O4*G*—H4*G*1···O6*G*^iv^	0.84	2.00	2.7884 (11)	155
O*W*1—H11···O2*F*	0.84 (2)	1.92 (2)	2.7522 (10)	172 (2)
O*W*1—H12···O4*F*^i^	0.83 (2)	1.94 (2)	2.7646 (10)	178 (2)
O*W*2—H21···O6*G*^iv^	0.81 (2)	2.09 (2)	2.8897 (10)	170 (2)
O*W*2—H22···O5*F*^v^	0.86 (2)	1.91 (2)	2.7682 (11)	176 (2)
O*W*3—H31···O*W*4*A*^i^	0.81 (2)	2.04 (2)	2.8424 (16)	176 (2)
O*W*3—H32···O7*M*^vi^	0.87 (2)	2.00 (2)	2.8559 (13)	169 (2)
O*W*4*A*—H41*A*···O2*G*	0.83 (2)	2.10 (2)	2.8615 (16)	151 (3)
O*W*4*A*—H42*A*···O*W*2^vii^	0.83 (2)	2.11 (2)	2.9390 (15)	174 (4)

Symmetry codes: (i) −*x*, *y*−1/2, −*z*+1; (ii) −*x*+1, *y*+1/2, −*z*+1; (iii) *x*−1, *y*, *z*−1; (iv) −*x*, *y*−1/2, −*z*; (v) *x*, *y*−1, *z*; (vi) −*x*, *y*+1/2, −*z*+1; (vii) *x*, *y*+1, *z*.
